# Understanding the performance and impact of public knowledge translation funding interventions: Protocol for an evaluation of Canadian Institutes of Health Research knowledge translation funding programs

**DOI:** 10.1186/1748-5908-7-57

**Published:** 2012-06-22

**Authors:** Robert K D McLean, Ian D Graham, Kwadwo Bosompra, Yumna Choudhry, Stephanie E Coen, Martha MacLeod, Christopher Manuel, Ryan McCarthy, Adrian Mota, David Peckham, Jacqueline M Tetroe, Joanne Tucker

**Affiliations:** 1 Evaluation Unit, Canadian Institutes of Health Research, 160 Elgin Street, Ottawa, ON, Canada; 2 Knowledge Translation Portfolio, Canadian Institutes of Health Research, 160 Elgin Street, Ottawa, ON, Canada; 3 School of Nursing, Faculty of Health Sciences, University of Ottawa, 451 Smyth Road, Ottawa, ON, Canada; 4 Institute of Gender and Health, Canadian Institutes of Health Research, University of British Columbia, 6190 Agronomy Road, Vancouver, BC, Canada; 5 School of Nursing, University of Northern British Columbia, 3333 University Way, Prince George, BC, Canada

## Abstract

**Background:**

The Canadian Institutes of Health Research (CIHR) has defined knowledge translation (KT) as a dynamic and iterative process that includes the synthesis, dissemination, exchange, and ethically-sound application of knowledge to improve the health of Canadians, provide more effective health services and products, and strengthen the healthcare system. CIHR, the national health research funding agency in Canada, has undertaken to advance this concept through direct research funding opportunities in KT. Because CIHR is recognized within Canada and internationally for leading and funding the advancement of KT science and practice, it is essential and timely to evaluate this intervention, and specifically, these funding opportunities.

**Design:**

The study will employ a novel method of participatory, utilization-focused evaluation inspired by the principles of integrated KT. It will use a mixed methods approach, drawing on both quantitative and qualitative data, and will elicit participation from CIHR funded researchers, knowledge users, KT experts, as well as other health research funding agencies. Lines of inquiry will include an international environmental scan, document/data reviews, in-depth interviews, targeted surveys, case studies, and an expert review panel. The study will investigate how efficiently and effectively the CIHR model of KT funding programs operates, what immediate outcomes these funding mechanisms have produced, and what impact these programs have had on the broader state of health research, health research uptake, and health improvement.

**Discussion:**

The protocol and results of this evaluation will be of interest to those engaged in the theory, practice, and evaluation of KT. The dissemination of the study protocol and results to both practitioners and theorists will help to fill a gap in knowledge in three areas: the role of a public research funding agency in facilitating KT, the outcomes and impacts KT funding interventions, and how KT can best be evaluated.

## Background

Around the world and across the spectrum of scientific and non-scientific goods and services, there is a desire for service and product provision to be informed by evidence. This desire has been made explicit within the realm of health research through the concept and ideal of evidence-based practice. However, the fact remains that health practice often lags behind knowledge and best practices established through health research [1,2]. To address this issue, efforts have been made to promote evidence-based practice and the use of research in practice. This is a concept that has become known by many names, including knowledge translation (KT) [3,4].

At the Canadian Institutes of Health Research (CIHR), Canada’s national health research funding agency, KT is defined as a dynamic and iterative process that includes synthesis, dissemination, exchange, and ethically-sound application of knowledge to improve the health of Canadians, provide more effective health services and products, and strengthen the healthcare system [5]. In more simple terms, KT at CIHR is about having research act as a driver of appropriate real-world applications. KT has been an important aspect of CIHR’s vision and work since the organization’s inception in 2000. In fact, to formalize the importance of KT to the organization, it was embedded in the CIHR mandate and written into the Parliamentary act that created CIHR as it now exists [6]. What this means in practice is that CIHR has written KT into its strategic plan, with a directive to accelerate the capture of the benefits of health research; created executive management roles and a unique branch of the organization devoted expressly to KT; and developed KT-specific funding mechanisms—the focus of the present evaluation protocol. The range of CIHR’s strategic activities and funding opportunities to address KT are designed to support not only KT science but also all of the elements of CIHR’s definition of KT (synthesis, dissemination, exchange, and ethically sound application of knowledge).

Paradoxically, and we believe to its disadvantage, the field of KT has lagged in what it is designed to address—the use of evidence to inform better products, services, and systems. Despite the fact that much evidence exists to support the need for KT, very little evidence exists that measures the performance and impact of KT interventions, especially when those interventions are funding mechanisms [4,7].

The evaluation research described in this protocol is designed to address this shortcoming of concern to practitioners and theorists alike. The aim of this study protocol, and the dissemination of its subsequent results, is to generate evidence in relation to the role of a public research funding agency in enabling/promoting KT, the outcomes and impacts of KT funding interventions, and how KT can best be evaluated. The study will investigate how efficiently and effectively the CIHR model of KT funding programs operates, what immediate outcomes these funding mechanisms have produced, and what impact these programs have had on the broader state of health research, health research uptake, and health improvement. The need for further research on the effectiveness of KT is especially imminent for the public funding agency, where KT interventions are designed to benefit the whole of society and are financed to do so by the taxpayer.

As very little evaluation has been conducted on the performance and promotion of KT, the study described in this protocol represents a unique approach to this complex task. The approach is grounded in the theoretical frameworks of both evaluation and KT. The remainder of the protocol presents an overview of this undertaking. The section that follows describes the approach to scoping an evaluation of CIHR KT interventions. The remaining sections outline the study methodology and provide a discussion of potential study implications.

## Scoping the study

### Evaluation purpose

Primarily, this evaluation study is designed to provide valid and insightful findings about the performance of CIHR’s KT programs for the purposes of program learning and future KT program development. The study will investigate how efficiently and effectively the CIHR KT funding programs operate, what immediate outcomes these funding mechanisms have produced, and what impact these programs have had on the broader state of health research, health research uptake, and health improvement.

The evaluation is also designed to meet CIHR’s requirements to Canada’s Treasury Board Secretariat (TBS) in order to demonstrate value for money in government spending*.* It therefore covers specific core TBS evaluation issues of program relevance and performance as described in the TBS policy suite ^a^. In the discussion section, we elaborate on the implications of designing the evaluation protocol to meet both our prospective program learning and development objectives, and retrospective accountability and reporting objectives.

The CIHR Act (Bill C-13) mandates CIHR to ensure that the translation of health knowledge permeates every aspect of its work [6]. An evaluation of all knowledge translation programs and activities at CIHR would therefore need to be broad in scope, be extremely resource intensive, and as such would likely only be able to provide very high-level findings. The intent of this evaluation is to provide evidence about the performance of CIHR’s overall KT strategy, but also to provide more detailed findings about the intricate factors surrounding the design and delivery of this strategy (*i.e.*, the targeted funding opportunities). Accordingly, the study outlined in this protocol is designed to address some of the key constraints and limitations of evaluating KT at CIHR including:

1. Ensuring that the evaluation is sufficiently targeted to investigate the idiosyncratic factors surrounding individual KT funding opportunities;

2. Ensuring the evaluation gathers data that reflect the operational definition of KT at CIHR (synthesis, dissemination, and ethically sound application of knowledge);

3. Ensuring that the evaluation is designed to gather data required by the Treasury Board while also collecting data that will address our objectives related to program learning and development;

4. Performing such research with a limited set of resources (particularly, time and financial constraints).

To address these considerations, a purposive sampling approach to selecting a set of KT funding programs was developed. Focusing on a comprehensive sample of funding programs enables the evaluation to provide detailed, precise, and useful findings at the program level. Triangulated and rolled-up, this data will provide an indication of the overall performance of CIHR’s KT strategy. Five CIHR KT funding domains were selected to be within the scope of this evaluation:

1. Knowledge Synthesis funding opportunity;

2. Partnerships for Health Systems Improvement (PHSI) funding opportunity;

3. Knowledge to Action (K2A) funding opportunity;

4. End of grant KT funding opportunities (Dissemination Events (DE) and the KT Supplement (KTS) programs)^b^;

5. KT research funding opportunity^c^.

These programs were selected and validated for inclusion based on two key criteria—program relevance and program materiality

### Program relevance

Programs were selected in order to provide full theoretical coverage of the four fundamental KT themes identified by CIHR management as representative of the purpose and concept of KT at CIHR. Details of these are provided in Table 1. The relevance review of KT programs was conducted through formal consultation with CIHR senior management and KT specific staff, and validated by external expert opinion ^d^.

**Table 1:** Relevance and materiality coverage of selected KT funding programs

**Selected funding program**	**KT area of focus**	**CAD(mil) - 2010-11 financial commitment**	**% of 2010-11 KT financial commitment**
Knowledge Synthesis	Synthesis; Integrated KT	1.76m CAD	10.6%
Partnerships for Health	Integrated KT	6.29m CAD	37.7%
System Improvement			
Knowledge to Action	Integrated KT	1.33m CAD	8%
DE and KTS	End grant KT	1.34m CAD	8%
KT research	KT science	n/a	n/a
**Total coverage**		**10.72m CAD**	**64.3%**

**Notes: 1)** Figures are based on finance coding for CIHR’s PAA 1.4.2.; **2)** KT research financial data is not included for the current period as money is not moved directly through PAA 1.4.2.; **3)** Programs not included in our evaluation as a whole represent 35.7% of PAA area spending (less Partnerships programs that were purposely removed) and for this period are: Reduce Health Disparities, Training Awards, CADRE, Clinical Research Initiatives, Health Research Community Awards, KT Awards, Mobility in Aging, Cochrane Canada, Youth and Public Engagement, Res Action Program in Dementia, Partnerships award, JBI, Journalism Awards, Evidence Review and Synthesis Centre, Canadian Knowledge Synthesis Network, Canadian Virtual Library Network.

### Program materiality

CIHR financial records were reviewed to assess the materiality of each KT program, and a risk-based approach to selecting programs was applied. The five selected domains represent approximately 65% of current financial commitments for the KT area at CIHR ^e,f^. Excluding the KT research funding opportunity within the open operating grants program (OOGP), the four programs also represent the four largest individual financial commitments provided through KT funding at CIHR.

### KT at CIHR and specific KT funding programs being examined

In this section, contextual details are provided on the concept of knowledge translation and the strategic approach to KT at CIHR. Subsequently, a description is provided of the funding programs that will be specifically examined through the evaluation study.

**Figure 1:**
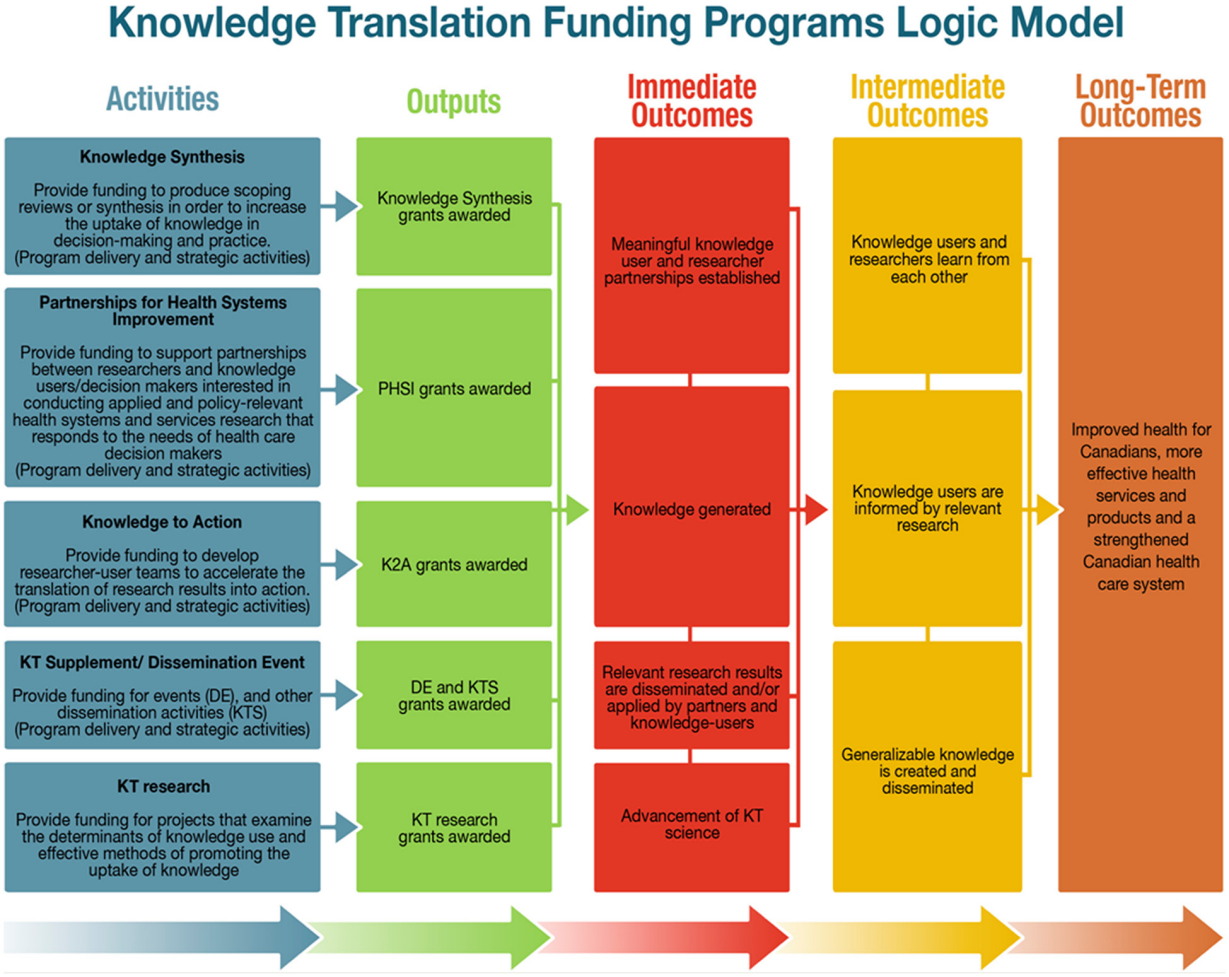
Knowledge translation funding programs logic model.

Figure 1 is a logic model ^g^ that was produced as a part of the planning of this evaluation. It provides a visual representation of the object of the evaluation.

### The concept of knowledge translation at CIHR

To promote/enable the concept of KT as defined by CIHR, there are four aspects supported by the organization: knowledge synthesis, integrated KT, end of grant KT, and KT science.

Synthesis is the contextualization and integration of research findings of individual research studies within the larger body of knowledge on the topic. It is a family of methodologies developed to determine what is known in a given area or field and what the knowledge gaps are. The underlying principle is the support of timely and accurate scientific knowledge being available to those who work in real-world settings requiring such evidence. Knowledge synthesis studies may be useful to policy-makers, industry, clinical, and medical practitioners, amongst others. In some cases, knowledge synthesis research can be conducted with the participation of non-traditional researchers throughout the research process. CIHR refers to the engagement of knowledge users in research as integrated KT (iKT).

Through iKT, stakeholders or potential knowledge users are engaged in the entire research process. By doing iKT, researchers and knowledge users work together to shape the research process by collaborating to determine the research questions, deciding on the methodology, being involved in data collection and tools development, interpreting the findings, and helping disseminate the research results. This approach is designed to produce research findings that are more likely to be relevant to and used by end users. This approach is similar to those described as collaborative research, participatory, action-oriented research, co-production of knowledge, and Mode 2 knowledge production.

End of grant KT describes the process where the researcher develops and implements a plan for making knowledge users aware of the knowledge that was gained during a project. End of grant KT includes the typical dissemination and communication activities undertaken by most researchers, such as KT to their peers through conference presentations and publications in peer-reviewed journals. End of grant KT can also involve more intensive dissemination activities that tailor the message and medium to a specific audience, such as summary briefings to stakeholders, interactive educational sessions with patients, practitioners, and/or policy makers, media engagement, or the use of knowledge brokers to name a few. The commercialization of scientific discoveries is another form of end of grant KT, but as a specific strategy it is not being explored in this study.

KT science or research (also known as implementation science) is the study of the process of KT and the use of knowledge. KT science explores the factors that facilitate and hinder the sharing of knowledge between creators and users. While it often addresses issues such as the efficacy of certain KT strategies, KT science may also involve the development of new KT theory or practice.

### Specific KT funding programs

#### Knowledge synthesis funding opportunity

Knowledge synthesis grants provide funding to researchers to produce scoping reviews or syntheses that meet the needs of decision makers or knowledge users in all areas of health. They support the concept that knowledge users should identify synthesis questions in collaboration with researchers so that the answers to these questions can inform policy, programs, and practice. They are also expected to increase the capacity of researchers to identify new, relevant avenues for exploration that have not yet been investigated that respond to decision makers’ and knowledge users’ needs [8]. Finally, because they are funded to be performed in an iKT format, synthesis grants are intended to promote the process of mutual learning between researchers and knowledge users.

First launched in 2004, CIHR invites all forms of knowledge synthesis. Qualitative, quantitative, and mixed methods approaches are accepted, as well as syntheses of knowledge gained through observation, testing, or reviewing of texts. Scoping reviews are also eligible; these are projects that explore the literature available on a topic, identifying the key concepts, theories, sources of evidence, and gaps in the research. They are often undertaken before a full synthesis when the literature is thought to be too vast or when there is suspicion that not enough literature exists to synthesize [8]. Because the knowledge synthesis funding opportunity is funded as iKT, applications to the funding opportunity undergo a merit review process.

Merit review is markedly different than typical CIHR peer review. The composition of iKT research teams and/or the nature of KT research projects require that merit review panels expand the traditional definition of ‘peer’ to include knowledge users whose expertise lies in the application of research. Because both researchers and knowledge users contribute to the production and the translation of research, merit review panel composition must reflect this, drawing members from both researcher and knowledge user communities. Each application is reviewed by at least one researcher and at least one knowledge user who assess potential impact and scientific merit; potential impact and scientific merit are weighted equally. Only those applications receiving a fundable score on both potential impact and scientific merit can be considered for CIHR funding [9].

### Resources

Knowledge synthesis competitions are launched twice a year by the CIHR Knowledge Translation Branch in partnership with various CIHR institutes and strategic initiatives, along with external partners. The maximum amount awarded for a synthesis is 100,000 CAD for one year. The maximum amount awarded for scoping reviews is 50,000 CAD for one year.

### Partnerships for health system improvement (PHSI) funding opportunity

The first CIHR PHSI competition was held in 2005 after it was transferred from Canadian Health Services Research Foundation. The PHSI funding program supports teams of researchers and decision makers/knowledge users interested in conducting applied and policy-relevant health systems and services research that respond to the needs of healthcare decision makers. Partnerships can be project specific (partners that the researchers identify themselves) or competition specific (CIHR negotiated competition partnerships). This funding opportunity requires pre-defined financial or in kind partner contributions [10]. PHSI grants are funded to be performed in an iKT format, and as such, this program uses a merit review process to evaluate applications.

### Resources

The maximum amount awarded by CIHR for a single grant is 400,000 CAD for up to three years (partnership contributions are in addition to the CIHR amount). A minimum of either 20% or 30%, depending on the province or territory, of the grant budget must come from external partner sources (*i.e.*, non-CIHR funds). There is no limit to partner contributions, and in-kind contributions are recognized, especially where they reflect meaningful collaboration that will increase the likely success of the project. It should be noted that funding and contributions may be received from stakeholders who are not members of the grant team.

### Knowledge to action (K2A) funding opportunity

K2A is designed to move knowledge into action by linking researchers and knowledge users and to increase the understanding of knowledge application through the process. By bringing both parties together, it is expected that research results will translate to actions that strengthen Canada's healthcare system and/or improve the health of Canadians. K2A also aims to support the development, implementation, and evaluation of cutting-edge KT research and approaches. Through this, the program establishes and strengthens common ground between the interests and expertise of the research community and the needs of knowledge users. Applicants can request funding to support partnerships, knowledge, and tools for implementation projects. This program was first launched in 2005. Because the K2A funding opportunity requires iKT, all applications go through a merit (not peer) review process.

### Resources

The maximum CIHR contribution is 100,000 CAD per year for up to two years. Applicants may increase funding for their proposal and further demonstrate the level of engagement of their partner(s) through cash or in-kind commitments, but a financial commitment from the partner is not a criterion for funding [11]. Applicants are encouraged to apply for a renewal of their grant if they plan to scale up their implementation project.

### End of grant KT funding opportunities: Dissemination events (DE) and the KT supplement (KTS)

The DE and KTS funding opportunities both support end of grant KT. DE is intended to provide support for meetings, and/or dissemination activities consistent with the mandate of CIHR and relevant CIHR institutes, initiatives, or branches. It supports the organization of events focused on the communication of health research evidence. The KT Supplement funding opportunity supports KT activities that follow implementation of a peer-reviewed grant/award where further dissemination is appropriate. Both DE and KTS applications undergo a peer review, rather than merit review, process.

Eligible activities for the DE funding opportunity include:

1. Education of groups such as patients, health professionals, community organizations, policy-makers, the general public;

2. Education of stakeholders regarding partnership best practices;

3. Knowledge dissemination that will inform practice, clinical care, policy and decision making;

4. Publishing articles in open access journals not budgeted for in other applications, as part of a broader dissemination strategy.

Eligible activities for the KTS funding opportunity include:

1. Development/maintenance/updating of websites;

2. Production and distribution of written materials in various formats;

3. Hiring of a knowledge broker or implementation facilitator/change agent;

4. Development of plain language summaries;

5. Development of knowledge exchange tools (*e.g.*, educational DVDs, decision support tools);

6. Dissemination of research results through specialized publications as part of a broader KT strategy, and;

7. Travel costs for a series of meetings/presentations (linkage and exchange activities) required to implement a broader KT strategy.

### Resources

These end of grant KT funding opportunities are non-renewable one-year grants. However, multiple grants can be awarded to the same candidate in the same calendar year. DE projects are funded up to 25,000 CAD, while KTS projects are funded up to 100,000 CAD [12].

### KT research funding opportunity within the OOGP

Funded KT research grants must be directed toward developing theory, evidence, and innovation to define the determinants, implementation, and uptake of health research evidence into practice. These include grants that aim to improve KT to consumers, health practitioners, and policy makers, to examine the role of organizations as KT vehicles, to determine how to improve knowledge uptake potential during the research process, to develop/evaluate KT tools and/or methods, and to contribute to KT theory and to improve knowledge uptake. KT research grants do not require knowledge user partners, although they are allowed, and are peer (not merit) reviewed.

### Resources

These OOGP competitions provide funding for up to five years and have no funding limit or specific requirements for team size or composition. Funding is allocated through the CIHR open operating grant budget rather than CIHR’s KT-specific budget. The Knowledge Translation Research committee is one of 53 standing committees on the OOGP.

## Methods

The following section outlines the investigation process that will be employed in this research. Each method of inquiry is described, and a brief preface about the process of design is included.

### Design and process

The study will employ a novel method of participatory, utilization-focused evaluation inspired by the notion of iKT. The study will use a mixed methods approach, drawing on both quantitative and qualitative data, and will elicit participation from stakeholder groups, including CIHR funded researchers, knowledge users, KT experts, TBS, and other health research funding agencies. The use of a mixed methods approach will be beneficial to uncovering significant detail about this complex intervention [13].

Utilization-focused evaluation is based on the idea that evaluations are only as efficacious as they are useful to their consumer(s). Patton [14] describes utilization-focused evaluation as being established on the premise that evaluations should be judged based on their actual use, and therefore, from planning to conclusion they should be conducted in the manner that is best adapted for intended end users. To us, stakeholders are much more likely to use this evaluation if they feel ownership over the evaluation purpose, process, and findings. By actively involving users throughout this evaluation, the foothold for use is being established and the utility of the evaluation is being continuously reinforced.

To realize this utilization approach, we have designed the protocol through a collaborative approach between multiple stakeholders, assembled in a research team that we have called the Evaluation Working Group. The Evaluation Working Group is chaired by CIHR’s Evaluation Unit, and included a broad spectrum of CIHR staff involved in developing, delivering, and evaluating the programs; these members represented key internal KT stakeholders with operational knowledge of KT program design and delivery (representing the KT Branch as well as a CIHR institute). The Evaluation Working Group also included an external (non-CIHR employed) researcher with KT expertise and funding who also serves as the chair of one of the merit review panels of interest. The combination of CIHR internal and external perspectives on the Evaluation Working Group ensured that the protocol development was grounded in the operational realities of CIHR and designed to provide appropriate input for program improvement purposes, while being attuned to practicalities of engaging with these programs and implementing funded projects on the program user side. The intent is to conduct the evaluation in a participatory fashion similar to what CIHR expects of applicants to its iKT funding programs. The Evaluation Working Group members involved in the design phase will remain involved through the entire research process. Indeed, conducting this research in a collaborative fashion will facilitate the utility of the evaluation through both process and product (or findings) benefits, and, as such, support the use of the evaluation [15]. *Ad hoc* participation from each group member is to be expected and encouraged, however, at five critical stages the entire group will meet to seek consensus and affirm their satisfaction and that their representation is upheld:

1. The design of the evaluation framework—this includes the study sampling process (described above), the design of the logic model, the design of research questions, and the selection of methods.

2. At the data collection phase the team will review all instruments and processes and take part in collection where appropriate.

3. When data are collected, the team will review findings from individual perspectives and then meet to form a group consensus on final interpretation and to learn how others reflections complement or detract from their own.

4. After measured contemplation of the findings, the group will consult to discuss best methods of developing an action plan to implement evaluation recommendations.

5. After measured contemplation of the findings, the group will consult to discuss best methods of dissemination to stakeholders, both external and internal.

### Evaluation questions

The evaluation will be focused on addressing a set of overarching questions regarding CIHR KT strategy through investigating funding program performance. In order to maximize the utility of the evaluation, these questions were developed collaboratively with the Evaluation Working Group.

The questions provide the overall direction for the evaluation; a series of detailed indicators and data sources designed to address these has been developed.

The overall evaluation questions are as follows:

1. What role is there for CIHR in enabling/promoting iKT research, synthesis, end of grant KT, and KT science?

2. To what extent are KT funding programs achieving their expected outcomes?

3. What factors facilitate or inhibit the achievement of funding program outcomes?

4. How effective is the mix of KT funding programs in achieving CIHR’s expected outcomes? (iKT, end of grant KT, KT science, synthesis)

5. To what extent have KT funding programs reached a broad and diverse range of knowledge users?

6. To what extent are KT funding programs being delivered as expected? Can any changes be made to program delivery in order to improve efficiency and effectiveness?

7. What would be the effect on CIHR-funded researchers and knowledge users if the KT program suite no longer existed? What would be the effect on the improvement of health, more effective health services and products, and the strengthening of the healthcare system?

8. What are the unanticipated outcomes, positive or negative, resulting from the KT funding programs?

The Evaluation Matrix in Table 2 provides full details of indicators and data sources

**Table 2:** Evaluation Matrix

**Evaluation questions**	**Indicators**	**Methods**	**Sources**
1. What role is there for CIHR in enabling/promoting synthesis, iKT, end-of-grant KT, and KT science?			
	● Theory and empirical evidence related to the role of a funding organization in the KT process	■ International environmental scan	■ 33 funding agencies from Tetroe *et al.* 2008 study
● Is the CIHR role consistent with the health needs of Canadians, the improvement of health products and services, and the strengthening of the Canadian healthcare system?	● Theory and empirical evidence related to the advantages and limitations of iKT research, end-of-grant KT, and KT science		
	● Degree of alignment of CIHR KT funding program suite with theory and empirical evidence of KT success strategies		
	● Organizational scan of comparable organizations nationally and internationally		
	● Expert opinion on the role of a funding organization in the KT process	■ External expert review	■ International KT expert panel
	● Expert opinion on the CIHR funding program mix		
	● Expert opinion on CIHR strengths, limitations, and strategic vision for KT funding programs		
	● Indications of incentive induced behaviour of researchers and knowledge users	■ Case studies	■ Exceptional funded projects
	● Indications of unique or innovative KT strategies employed		
	● Application pressure (total applications per funding program)	■ Document and EIS data review	■ EIS application records
	● Ratio of researchers funded *versus* applied		■ CIHR guiding documents
	● Ratio of researchers funded *versus* fundable but not funded		■ Government of
	● Degree of alignment with CIHR mandate and strategic vision		■ Canada documentation
	● Degree of alignment with the government of Canada’s plans and priorities? (*i.e.* SandT Strategy)		
2.To what extent are KT funding programs achieving their expected outcomes?	● Indications of immediate, intermediate, and long-term outcomes	■ Surveys	■ Funded researchers/knowledge users
● To what extent are immediate outcomes being achieved?			
● To what extent are intermediate outcomes being achieved?			
		■ Key informant interviews	■ Funded researchers/knowledge users
		■ Case studies	■ Exceptional funded projects
	● The number of grants awarded by each program	■ Document and EIS data review	■ EIS application records
	● # of partnerships created (iKT)		■ End of grant reports
	● Comparison of application pressure across funding programs		
	● Indications of intermediate and long term outcomes		
	● Degree of alignment of KT funding progam suite with theory and empirical evidence of KT success strategies	■ International environmental scan	■ 33 funding agencies from Tetroe *et al.* 2008 study
		■ External expert review	■ International KT expert panel
3. What factors facilitate or inhibit the achievement of outcomes?	● Indication of influence on program theory from:: internal program processes; external environmental factors; strategic level factors; program delivery level factors	■ Surveys	■ Funded researchers/knowledge users
		■ Key informant interviews	■ Funded researchers/knowledge users
		■ Case studies	■ Exceptional funded KT projects
		■ Document and EIS data review	■ EIS application records
			■ Final reports
	● Program delivery level factors		
4. How effective is the mix of funding programs in achieving CIHR’s expected outcomes? (iKT, End of grant-KT, KT Science, Synthesis)	● Perceptions of suitability of program mix for promoting/enabling effective KT	■ Key informant interviews	■ Funded researchers/knowledgeusers
		■ Surveys	■ Funded researchers/knowledge users
	● Profiles of pathways to program outcomes	■ Case studies	■ Exceptional funded KT projects
	● Degree of alignment of CIHR KT funding program suite with theory and empirical evidence of KT success strategies	■ External expert review	■ International KT expert Panel
		■ International environmental scan	■ 33 funding agencies from Tetroe *et al.* 2008 study
5. To what extent have KT funding programs reached a broad and diverse range of knowledge users?	● Number and type of knowledge users included per iKT grant	■ Document and EIS data review	■ EIS application records
			■ Final reports
	● Perceptions of meaningful partnerships having been established	■ Surveys	■ Funded researchers/knowledge users
		■ Key informant interviews	■ Funded researchers/knowledge users
		■ Case studies	■ Exceptional funded KT projects
6. To what extent are KT funding programs being delivered as expected? Can any changes be made to program delivery in order to improve efficiency and effectiveness?	● Indications of efficiency and effectiveness in the conversion of program activities into program outputs	■ Document and EIS data review	■ EIS application records
		■ Surveys	■ Funded researchers/knowledge
	● Identified success and challenges of the merit review process		■ users
		■ Key informant interviews	■ Funded researchers/knowledge users
		■ Case studies	■ Exceptional funded KT projects
7. What would be the effect on CIHR-funded researchers and knowledge users if the KT funding program suite no longer existed? What would be the effect on the improvement of health, more effective health services and products, and the strengthening of the healthcare system?	● Perceived impact of absence of future KT funding on funded researchers, knowledge users, and KT outcomes	■ Surveys	■ Funded researchers/knowledge users
	● Perceived future directions for funded researchers, knowledge users, and KT outcomes in the absence of KT funding	■ Key informant interviews	■ Funded researchers/knowledge users
	● Use of alternative funding sources by KT funded teams (leveraging)	■ Case studies	■ Exceptional funded KT projects
	● Use of alternative funding sources by KT researchers and knowledge users not funded by CIHR (Knowledge User partners)	■ EIS	■ EIS application records
	● Organizational scan of similar organizations nationally and internationally		■ Final reports
		■ International environmental scan	■ 33 funding agencies from Tetroe *et al.* 2008 study
8. What are the unanticipated outcomes, positive or negative, resulting from the KT funding programs?	● Identified unintended outcomes of KT funding programs	■ Document and EIS data review	■ EIS application records
			■ Final reports
		■ Surveys	■ Funded researchers/knowledge users
		■ Key informant interviews	■ Funded researchers/knowledge users
		■ Case studies	■ Exceptional KT funded projects
		■ International	■ 33 funding agencies
		■ environmental scan	■ from Tetroe *et al.* 2008 study
		■ External expert review	■ International KT expert panel

Note: Indicators and sources presented in this matrix are not static. As the research process progresses, the Evaluation Working Group will be attuned to new information that may create the need for review

### Methods of investigation

To ensure that findings are robust and that valid conclusions can be drawn about the performance of the programs, the evaluation will use multiple methodologies and draw on both quantitative and qualitative evidence. A range of methods will be employed to capture a wide diversity of data, namely: an international environmental scan of health research funding agencies; a document review and CIHR’s Electronic Information System (EIS) data review; in-depth key informant interviews; quantitative surveys; case studies; and an external expert panel discussion. A range of quantitative and qualitative data analysis techniques will be used to interpret each source of data, and are described under each heading below. To ensure rigour, our analysis will triangulate findings from all methods to inform study conclusions.

### International environmental scan

A review of organizations from a range of countries that provide KT research funding will be conducted in order to gather information regarding the how they fund KT and what might be considered best practices in the field. The environmental scan will focus predominately on the first evaluation question (CIHR’s role in enabling/promoting KT).

The scan will be formulated as an update and expansion on a 2008 publication by Tetroe *et al.* entitled ‘Health research funding agencies' support and promotion of knowledge translation: an international study.’ The scan will use the same sample frame of organizations. A review of each agency’s website and public documentation will be performed, and follow-up semi structured interviews with a KT contact person and an evaluation contact person from each agency will be pursued. Completed data templates will be sent to each organization for validation.

The environmental scan will provide context and evidence surrounding the role of a funding agency in KT processes, the known successes and limitations of various KT funding programs, as well as KT evaluation. A comprehensive review of empirical evidence related to these three subjects will allow for the development of a contextual base for the remainder of data collection phases, and will situate CIHR in comparison to similar organizations around the world. This component of the evaluation will also yield important insights that will contribute to global literature on KT science, specifically filling a knowledge gap in relation to public funding interventions for KT and their evaluation. The Evaluation Working Group will be engaged throughout the process.

### Document review and electronic information system data review

A document and data review will be a significant source of information for this study in order to address each of the evaluation questions. Documentation to be reviewed will include key CIHR publications and Government of Canada publications related to the study topic. The Evaluation Working Group will work closely to identify and locate key documentation that is pertinent to each stakeholder group (*i.e.*, CIHR staff, KT researchers and knowledge users, program users, TBS).

CIHR’s EIS data will be used to obtain and analyze applicable information concerning KT funding program applicants. Where necessary, the CIHR Finance Unit will be approached for financial datasets.

Although initial data and document mining will be based on identified questions and indicators, the document and EIS data review will be ongoing throughout the data collection phase. As such, the process will be reactive to the most current discoveries and suggestions from other lines of investigation and provide an ongoing source for triangulation of findings.

### Key informant interviews

Semi-structured in-depth interviews with researchers and knowledge users awarded CIHR KT grants will be conducted to gather information on key stakeholders’ perceptions and experiences with CIHR KT funding programs. The qualitative data gathered through the interviews will provide important context to issues explored through surveys and data review. Qualitative data are, for example, particularly useful in understanding why participants hold particular views, or when seeking to understand a more complex interaction or procedure.

Interviews will be conducted with two discrete sample groups, in order to capture a diverse and balanced view of performance. Researchers and knowledge users will be interviewed, where possible from the same funded KT project. The combination of the two perspectives from a single project will be used to elicit the shared and distinct opinions of the two. It is anticipated that this approach will unearth robust detail. Up to 30 interviews will be conducted, or until saturation is reached, with funded researchers (n ≈ 15 interviews) and funded knowledge users (n ≈ 15 interviews).

Interviewees will be selected on the basis of the following criteria: funding program used, experience with CIHR/CIHR KT research, research area (CIHR research pillar *i.e.*, biomedical, clinical, health services, social, cultural, environmental, and population), Canadian official language (French or English), and geographic location.

The interviews will be conducted by telephone and versioned interview guides will be developed for the researcher and knowledge user interviews. The tailoring of guides to each stakeholder group will illuminate differing experiences and perspectives. Interviews will be designed to be approximately 45 to 90 minutes in length. All interviewees will be afforded full confidentiality in their responses, and collected notes and recordings will be managed in accordance with the federal Privacy Act. Interview data will be coded and analysed using NVivo software; data will also undergo review by the Evaluation Working Group to identify and recount key subjective experiences of the interviewees using constant comparative analysis (*i.e.*, taking data and comparing it to others that may be similar or different).

### Quantitative survey

A quantitative survey will be used in order to gather more generalizable information related to funding program performance. The quantitative survey will be launched following the key informant interviews. This structure will allow for specific lines of investigation in the survey to be informed by interview responses. Furthermore, it allows for a test of language and question framing issues to be performed in the interactive interview setting, and thus, for the survey to be framed in the most appropriate way for the target population. The survey design was developed through an iterative feedback process with the Evaluation Working Group.

Funded researchers will be surveyed, as well as a counterfactual group ^h^ of researchers funded through CIHR’s non-KT funding opportunities. The partial coverage of the CIHR KT funding programs (*i.e.*, not everyone who is eligible applies to the programs) allows for comparison between a group of KT funding program applicants and non-applicants, and will help identify program effects and impact.

Surveys will be administered to the full population of recipients of each of the five KT funding programs (n ≈ 600). The comparison (counterfactual) group will be selected based on responses to CIHR’s research reporting instrument, which was recently piloted. This is CIHR’s general end of grant reporting tool, and it has been administered to recipients of open operating grants (not these KT programs).

The survey will be hosted online, and participants will be invited to take part by email. In order to minimize burden on respondents, surveys will be designed to take less than 30 minutes to complete. Questionnaires will be developed with reference to overarching evaluation questions and will be tailored to each funding program, but will also have commonalities to allow for valid comparisons across groups. Respondents will be assured of full confidentiality. The survey will be undergo a pre-test period to allow for corrections and streamlining.

Where appropriate, we will draw on the design of questionnaires used in previous evaluations of KT programs, both within CIHR and externally. Data collected from surveys will be analysed appropriately (*i.e.*, bivariate and multivariate cross examination where statistically robust) using SPSS. Results will be reviewed and interpreted by the Evaluation Working Group.

### Case studies

A total of five case studies will be undertaken in order to investigate and illustrate links between funding program activities and outcomes. A case study method of inquiry will provide empirical data regarding occurrences of KT and a frame of context surrounding the setting where this trend and/or occurrence took place. The case studies will employ a pathway case analysis format. This analysis method is useful when the result of an intervention is known (in our case, an exceptional demonstration of expected KT outcomes), and the starting point of the intervention is relatively similar (in our case, a funded KT project). The pathway analysis allows for the investigation of causal factors of influence affecting the intervention [16]. Case study investigation will supplement other lines of evidence by providing rich and detailed accounts of the knowledge translation process.

One case study will be conducted for each of the five KT funding programs. The selection of one successful funded project per program will be an interactive process engaging the Evaluation Working Group Projects will be selected that demonstrate exceptional instances of KT outcomes, so that lessons can be drawn about what pathway factors lead to success.

Case studies will be developed based on a common semi-structured interview process with a funded team of researchers and knowledge users, a review of project documentation, and site visits where appropriate. A common approach to data collection will allow for the analysis of similar issues and questions across varied projects and the meaningful comparison of findings. The approach will be developed through consultations of the Evaluation Working Group, and case study drafts will be reviewed by all group members.

With this in mind, the design of the case study research will not be a ‘checklist’ approach built against pre-determined indicators. The approach will provide ample flexibility for the documentation of not only the KT process within a project, but equally important, the environment in which the process occurred. Documenting this environment will provide valuable context to the KT processes and to understanding their success.

### External expert review panel discussion

An expert review will be undertaken in order to provide expert insight into the CIHR KT funding programs and to provide an arm’s length assessment of the evaluation and its findings. The perceived position of CIHR as a Canadian and global leader in KT provides a unique opportunity for attracting the interest of leading subject area experts to provide advice and opinion on CIHR funding program strategy. At the same time, this position of leadership necessitates critical review by the most accomplished of specialists. Reviewers invited to participate the panel will be KT specialists of international repute, the majority being from countries other than Canada; some Canadian experts who have received funding from CIHR may be included. No CIHR staff will be on the expert panel in order to reduce bias in interpreting the data.

The study will be designed to provide a forum for discussion between leading KT area experts. The panel will also review the data, analysis and interpretations of the Evaluation Working Group, and be asked to comment on the rigour and accuracy of the evaluation. More precisely, our deliberative approach will involve an expert group, a series of iterations where information is collected, processed by a moderator, and returned to the Evaluation Working Group members for further analysis based on collective input. The process will allow for inferences to be drawn by leading thinkers in the field.

Issues explored in the study will relate to CIHR KT funding programs as well as the wider CIHR KT strategy. Primarily, evaluation questions one and two will be the major focus of this line of investigation. However, the study will be timed to conclude the data collection phase so that key issues arising from each line of evidence can be explored in greater depth through the method.

Data collection sessions and communications will be moderated by the Evaluation Working Group in order to ensure neutrality. In order to ensure meaningful results, the Evaluation Working Group will also be fully engaged in the design of the instrument and the selection of participants.

### Analytic approach: Triangulating data from multiple sources

The five methods of data collection described above fit together as part of a data triangulation strategy. The components were designed and will be sequentially carried out in order to iteratively influence the design of subsequent components; in this way, the protocol cumulatively builds on each data type. These multiple sources of data will serve to uphold rigour in our analysis because findings from each component will be cross-checked for consistency and investigated where discrepancies arise.

### Reporting approach

The study protocol (this document), is the first major report stemming from the planned evaluation. The purpose of this report is to encourage the process of this evaluation to be shared and criticized, and thus, to encourage learning about best practices in the evaluation of KT.

Final results of the study will be reported on in aggregate form in a final evaluation report. Once finalized, this evaluation report will be submitted to TBS and made publicly available on CIHR`s website.

Additional publications, presentations, and other dissemination items/events will be crafted wherever possible and appropriate, and may be prepared for any of the data collection methods individually or in combination. Given that a key driver of this research is the lack of knowledge surrounding KT effectiveness, optimal approaches to funding KT, and the evaluation of KT, reporting on the process and results of this study becomes an essential purpose. We also plan to write a paper on our experience conducting the evaluation in this way.

### Ethical considerations

The project is being performed under the auspices of CIHR’s requirement to evaluate its expenditures as is mandated for all public organizations in Canada’s Treasury Board Secretariat *Policy on Evaluation*. As a part of our relationship with TBS, the research undertaken in the evaluation of federal public expenditures is ethically authorized under the *Values and Ethics Code for the Public Service* and the *Privacy Act*. Even as a federal funding agency responsible for the development and ongoing management of the *Tri-Agency Policy on Ethical Research* our evaluation research is ethically authorized under the aforementioned federal policy, code, and act without Research Ethics Board review.

However, the study has also been approved by the Ottawa Hospital Research Ethics Committee. Full measures will be taken to uphold research ethics in accordance to our relationship with TBS and the Ottawa Hospital Research Ethics Committee conditions.

Funding for the evaluation will come from that portion of the CIHR corporate budget allocated to the systematic and regularly scheduled evaluation of the CIHR grants and awards programs.

## Discussion

In addition to the routine challenges of undertaking a complex evaluation with multiple data sources and stakeholders, there may be some unique ones related to this evaluation. In particular, an evaluation that actively engages program owners may raise concerns about its independence. Another potential challenge relates to conflicts that may arise during the evaluation between members of the Evaluation Working Group, and how they should be resolved.

To minimize the risk of any biases being introduced in the evaluation, we built in a number of checks and balances in our protocol design. However, it should be noted that CIHR’s current approach to evaluation does involve engagement of the program owners (*i.e.*, staff who develop and administer funding opportunities) in reviewing data and recommendations. These program-owners may have vested interests in the program, and may wish to influence the recommendations. CIHR’s governance structure is designed to minimize the occurrence of this. An oversight committee exists that reviews all evaluation plans and reports to ensure that the appropriate methodologies are used, analysis is undertaken, and that recommendations are supported by evaluation data. This process is in place for the KT evaluation we are proposing to undertake.

Another way we have designed the evaluation to minimize the introduction of bias is the expert review panel. This panel will review the data and the interpretations developed by the Evaluation Working Group and offer an independent opinion on the analysis. Ultimately though, it is the integrity of the people involved that must be relied upon to ensure the evaluation is undertaken in a rigorous and transparent fashion. CIHR evaluators and other staff operate under a government-wide code of conduct and perform their duties with professionalism and due diligence, and the Evaluation Working Group members have all be sensitized to reflect on their individual potential biases.

While disagreements or tensions among the Evaluation Working Group are not anticipated given the good working relationships already existing between the members of the group, we do recognize that during the course of the evaluation, differences of opinion may arise given the different perspective of group members. We have agreed that ongoing open and frank discussions during our meetings will be a mechanism for addressing any conflicts. Should the Evaluation Working Group fail to be able resolve conflicts on its own, these issues will be brought to the oversight committee.

In sum, the protocol and results of this evaluation will be of interest to those engaged in the theory, practice, and evaluation of KT. The dissemination of the study protocol and results to both practitioners and theorists will help to fill a gap in knowledge in three areas: the role of a funding agency in facilitating KT, the outcomes and impacts of KT funding interventions, and how KT can best be evaluated.

## Endnotes

^a^For further detail on the TBS policy suite see: http://www.tbs-sct.gc.ca/cee/pol-eng.asp

^b^ DE and KTS funding opportunities are distinct programs designed to support end of grant KT.

^c^ KT research projects are funded through CIHR’s Open Operating Grant program.

^d^ An external expert on KT (MM) was involved in each step of the evaluation planning process and sat as a member of the evaluation working group.

^e^ This excludes partnership programs. The Partnerships Branch of CIHR was consulted as a part of the planning phase of this evaluation. Results of this consultation indicated that the evaluation of partnerships programs within the portfolio would not yield useful results for program management in the context of a KT level evaluation.

^f^ Commercialization programs are not included in the evaluation as they fit into a separate envelope of CIHR KT focus and strategy.

^g^ A logic model is an illustrative tool used to provide a simple, visual representation of the theory of change of an intervention.

^h^ The measurement of a counterfactual group is a natural and social science method of discerning causality. In our case, a group of program participants will be compared to a group of individuals who did not participate in the program. Comparison between the two groups against appropriate evaluation questions will allow for the claims about the attribution that programs have had toward responses to these questions. It should be noted that sampling techniques and the statistical significance of results must be carefully considered in this method.

## Logic model narrative

KT has two bodies of officers, strategic leads and program officers. The activities of the KT suite of programs at CIHR have many similarities and therefore, are described together, except for A5.

### Strategic leads

Strategic leads research, design and implement CIHR’s KT strategies. These activities include:

• Designing programs and funding opportunities, including the formulation and modification of program regulations and processes

• Designing literature

• Offering training opportunities

• Conducting Research in the area of KT

• Running KT events

• Promotion and communication activities

### Program officers

• A1-A4:

• Administration, including application processing, organization of peer or merit review, notification and post-award notification

• MPD and KTR programs have peer review competition

• Knowledge Synthesis, PHSI and K2A programs have merit review competitions

• Monitoring of performance of program activities and outcomes

### KTR review panel (A5)

• The KT research panel reviews all KT related applications received in Open Operating Grants competitions

### Outputs

• O1 - Knowledge synthesis grants awarded:

• Knowledge synthesis grants provide funding to researchers who intend to produce scoping reviews or syntheses that meet the needs of knowledge users in all areas of health.

• Grants are expected to increase the capacity of researchers to identify new, relevant avenues for exploration that have not yet been investigated that respond to decision makers/knowledge users’ needs.

• O2 - PHSI grants awarded:

• Researchers and decision makers enter into partnerships to conduct applied and policy-relevant health systems and services research that respond to the needs of healthcare decision makers.

• Partnerships can be project specific (partners that the researchers identify themselves with and with whom they negotiate) or competition specific (CIHR negotiated competition partnerships). Partners providing financial assistance are not required to be team members.

• O3 - K2A grants awarded:

• Grants are awarded to move knowledge into action by linking researchers and knowledge users located in the same community or region.

• The development, implementation and evaluation of cutting-edge KT research and approaches are also supported.

• O4 - DE and KTS grants awarded:

• Grants are awarded to support meetings, and/or dissemination activities consistent with the mandate of CIHR and relevant CIHR Institutes, Initiatives or Branches.

• Dissemination Events support the organization of events focused on the communication of health research evidence.

• The KT Supplement supports KT activities that follow a CIHR grant/award where further dissemination is appropriate.

• O5 - KT research grants awarded:

• Grants fund research directed toward developing theory, evidence and innovation to define the determinants, implementation and uptake of health research evidence into practice.

### Immediate outcomes

• IMM 01 - Meaningful knowledge user and researcher partnerships established

• Partnerships between researchers and knowledge users are established, with knowledge users active in the research process

• IMM 02 – Knowledge generated

• Funded projects result in the generation of knowledge.

• Quality knowledge includes syntheses of related research findings

• IMM 03 - Relevant research results are disseminated and/or applied by partners and knowledge users

• Researchers and knowledge users work together to address relevant research questions and to exchange and apply knowledge to solve health and health system problems. This results in research findings that are relevant to the knowledge user partners.

• IMM 04 - Advancement of KT science

• KT-funded grants advance the knowledge of KT in areas such as new approaches to KT, innovative KT tools, research into new strategies for facilitating the translation of findings into practice, *etc.*

### Intermediate outcomes

• INT 01 - Knowledge users and researchers learn from each other: Researchers/knowledge users are active in post-research knowledge translation activities. Knowledge users are well informed by relevant research.

• By bringing both researchers and knowledge users together, it is expected that research results will translate to actions that strengthen Canada's healthcare system and/or improve the health of Canadians

• KT capacity is developed, increasing the KT expertise in Canada

• INT 02 - Knowledge users are informed by relevant research: Application of research findings by knowledge users

• The inclusion of knowledge users within the research process fosters greater ownership among knowledge users. This results in improved rates of application by knowledge users.

• Application includes the awareness of findings among knowledge users, influence/inclusion of research findings in policy decisions, adoption of findings into practice, *etc.*

• INT 03 - Generalizable knowledge is created and disseminated

• Research results from funded studies are made widely applicable and disseminated outside the sphere of knowledge users and researchers directly related to the project.

### Long term outcome

Ultimately, the KT suite of funding programs are intended to facilitate the translation of research into application in society at large, resulting in the improved health for Canadians, more effective health services and products and a strengthened Canadian health-care system. The KT suite of funding programs are aimed to work together to improve KT capacity in Canada, improve knowledge of KT and integrate both researchers and knowledge users in the research process, improving the relevance and timeliness of research findings.

## Abbreviations

CIHR: Canadian Institutes of Health Research; KT: Knowledge Translation; TBS: Treasury Board Secretariat; PHSI: Partnerships for Health Systems Improvement; K2A: Knowledge to Action; iKT: Integrated Knowledge Translation; DE: Dissemination Events; KTS: Knowledge Translation Supplement; OOGP: Open Operating Grants Program; EIS: Electronic Information System.

## Competing interests

RKDM is an Evaluator at CIHR. As Principal Evaluator, RKDM is responsible for the production of evaluation reporting for program development and meeting requirements of TBS. IDG is the Vice President of Knowledge Translation and Public Outreach at CIHR. In this role and in the role of Principal Evaluation User, IDG is ultimately responsible for the KT funding programming being evaluated. KB is an Evaluator at CIHR. YC is Senior Knowledge Translation Specialist at CIHR. SEC is Knowledge Translation Manager at CIHR’s Institute of Gender and Health. MM is chair of CIHR’s K2A merit review panel. CM is a Junior Evaluator at CIHR. RM is Director of CIHR’s KT Branch. AM is Manager, KT Initiatives at CIHR. DP is Manager of CIHR’s Evaluation Unit. JMT is a Senior Advisor at CIHR. JT is a Junior Evaluator at CIHR.

## Authors' contributions

All authors contributed to study conception and participated in critically appraising and revising the intellectual content of the manuscript. All authors read and approved the final manuscript. RKDM is Principal Evaluator for this study. IDG is Principal Evaluation User for this study.
